# Perspectives of *Homo sapiens* lifespan extension: focus on external or internal resources?

**DOI:** 10.18632/aging.102981

**Published:** 2020-03-27

**Authors:** Vladimir P. Skulachev, Gregory A. Shilovsky, Tatyana S. Putyatina, Nikita A. Popov, Alexander V. Markov, Maxim V. Skulachev, Victor A. Sadovnichii

**Affiliations:** 1 Belozersky Institute of Physico-Chemical Biology, Lomonosov Moscow State University, Moscow 119991, Russia; 2 Institute for Information Transmission Problems, Russian Academy of Sciences, Moscow 127051, Russia; 3 Faculty of Biology, Lomonosov Moscow State University, Moscow 119991, Russia; 4 Faculty of Bioengineering and Bioinformatics, Lomonosov Moscow State University, Moscow 119991, Russia; 5 Paleontological Institute, Russian Academy of Sciences, Moscow 117997, Russia; 6 Faculty of Mechanics and Mathematics, Lomonosov Moscow State University, Moscow 119991, Russia

**Keywords:** life expectancy, aging, neoteny, anti-aging drugs, demography

## Abstract

*Homo sapiens* and naked mole rats (*Heterocephalus glaber*) are vivid examples of social mammals that differ from their relatives in particular by an increased lifespan and a large number of neotenic features. An important fact for biogerontology is that the mortality rate of *H. glaber* (a maximal lifespan of more than 32 years, which is very large for such a small rodent) negligibly grows with age. The same is true for modern people in developed countries below the age of 60. It is important that the juvenilization of traits that separate humans from chimpanzees evolved over thousands of generations and millions of years. Rapid advances in technology resulted in a sharp increase in the life expectancy of human beings during the past 100 years. Currently, the human life expectancy has exceeded 80 years in developed countries. It cannot be excluded that the potential for increasing life expectancy by an improvement in living conditions will be exhausted after a certain period of time. New types of geroprotectors should be developed that protect not only from chronic phenoptosis gradual poisoning of the body with reactive oxygen species (ROS) but also from acute phenoptosis, where strong increase in the level of ROS immediately kills an already aged individual. Geroprotectors might be another anti-aging strategy along with neoteny (a natural physiological phenomenon) and technical progress.

## INTRODUCTION

The nature of the selection factors underlying the evolution of aging remains controversial [1-3]. Many specialists in evolutionary gerontology support a set of ideas called the “evolutionary theory of aging” [1, 2]. This theory is based on the idea that the selection efficiency decreases with age. It is also assumed that vitality and fertility are high in youth at the cost of reduced fitness at later ages [4, 5]. An alternative view is that programmed aging and death may be favored by some kind of selection [3, 6–16].

A subsequent theoretical experiment called the “Fable about Fox and Hares” was suggested by one of the authors of this paper (VPS) [17]. Two young hares differing “intellectually” have equal chances to escape from a fox since both of hares are running faster than a fox. However, with age, the clever hare acquires some advantage, which becomes of crucial importance when the running speed of hares lowers to that of a fox. Now, the clever hare has a better chance to escape and, hence, to produce clever leverets than the stupid hare. Such an effect becomes possible due to age-dependent lowering of the running speed as a result of the operation of an aging program. This will facilitate the selection for cleverness. Recently, attempts have been made to analyze the fable by computer modeling [18, 19].

The retardation of the operation of chronic aging programs in humans [13, 20–23] and in naked mole rats [13, 24–26] and the acute senile phenoptosis in the nematode *Caenorhabditis elegans* [12, 14, 15, 27–29] strongly supports the idea that programmed aging plays at present an important role in the abovementioned three animal species.

It is clear that the longevity in highly social mammals, such as *H. glaber* and humans, is partly due to neoteny, i.e. prolongation of youth and retardation of aging. The aging in both species cannot at present promote evolvability: in *H. glaber* – due to its hierarchy where only the “queen” and her “husbands” participate in breeding, and in humans – due to the rapid technological progress that in fact replaces the very much slower biological evolution [13]. As to *C. elegans,* the operation of the aging programs is clearly needed for the production of yolk [29] rather than for the stimulation of natural selection.

This paper is devoted to a comparative analysis of the survival curves of humans and related primates and the possible role of neoteny and other mammalian anti-aging programs in lifespan (LS) prolongation.

## Possible mechanisms of changes in the survival curves

Various characteristics of LS have been used for survival curve analysis. One important factor is the heterogeneity of mortality causes, which can be divided into internal factors (intrinsic mortality) and external influences (extrinsic mortality) [30–33].

### Terminal-to-average mortality ratio

Jones et al. [33] compared the mortality of Ache Indians, Swedish females (born in 1881), Japanese females (died in 2009), two species of nonhuman primates and 40 other species of animals and plants. The authors analyzed the interval between (i) the onset of sexual maturation and (ii) “terminal” age corresponding to the 95% mortality of the original sexually mature cohort (LS_95_). Their criterion (mortality at “terminal” age versus the average mortality rate for the entire study period) led to several obviously erroneous conclusions. For instance, the great tit *Parus major* was assigned to the non-aging species and placed next to hydra. However, *P. major* just does not have time to grow old owing to a high age-independent mortality. LS_95_ for the pine *Pinus silvestris* was as short as 30 years due to the high mortality of the young trees. Tortoise was declared as the most non-aging animal, while crocodile was in the middle of the list. Moreover, long-lived humans (Japanese and Swedes) and animals (southern fulmar) were classified as the species with the most pronounced aging, despite having a long period of a negligibly low mortality rate.

### Coefficient of variation of lifespan (CV_LS_)

It is assumed that we can expect a high variance in LS (i.e., high CV_LS_ values) in the studied population when the degree of genetic regulation of aging is low (i.e., poorly controlled LS) and the rate of background “extrinsic” mortality is high. On the other hand, the LS variability in the population should be low when LS is stringently regulated by the genotype. In this case, it can be assumed that the “true” value of the species LS will be the value in the case of a smaller relative variation of LS. Upon great variation, it can be assumed that the external causes (predators, hunger, etc.) have a higher impact on mortality, while the genome-encoded mechanisms of longevity assurance play a secondary role.

N. Gavrilova, L. Gavrilova, F. Severin and V. Skulachev [34] compared the coefficient of variation (CV) of the parameter determined by the female *H. sapiens* development program (puberty age) and the aging-related parameters (menopause and death age). The data from the National Survey of the Midlife Development in the U.S. (MIDUS) were used. It has been found that the CV = 8–13% for the age of the onset of puberty, CV = 7-11% for the age of the onset of menopause, and CV = 16–21% for the age of death. Two results of these calculations are noteworthy. (A) The CV values for puberty and menopause coincide, which is predicted by the programmed aging concept since both events should be controlled by an ontogenetic clock. The alternative concept considering aging as a result of stochastic accumulation of damage cannot explain the above coincidences of CVs for puberty and menopause. (B) Variability of the age of death was two times higher than for puberty and menopause. Such relationships are not surprising since, in addition to programmed aging, there are external death factors that increase the variability of death.

Thus, the programmed aging has been demonstrated with a statistical method.

CV_LS_ estimates based on the Jones et al. [33] data from people >10 years old were 16.2% ± 0.1% for the Japanese females, 37.1% ± 0.3% for the Swedish females, and 54.5% ± 0.3% for the Ache Indians. For comparison, CV_LS_ was 59.2% ± 0.2% in >7-year-old chimpanzees [puberty age] and 53.9% ± 0.1% in baboon females in 4 years old [35]. At the same time, the LS_95_ was 24 years for the baboon females, 49 years for the chimpanzee females, 81 years for the Ache Indians, 89 years for the Swedish females and 102 years for the Japanese females [33]. It is important that Ache hunter-gatherers have, as a rule, minimal (near zero) dependence on modern medicine and do not use any commercial products in their diets [36].

These data are consistent with the idea that the observed variation in human LS in less developed societies is determined to a larger extent by unpredictable extrinsic factors, while the genetic component of variation becomes more prominent in more developed societies.

The human LS steadily grows in a series of generations. For instance, Oeppen and Vaupel [37] showed that the human life expectancy at birth (e_0_) was growing almost linearly from 1840 to 2000 in developed countries, with an increase of approximately 3 months per year. This growth is due to a rapid steady decline in mortality at all ages [38], with the exception of the mortality of centenarians [39].

The analysis of the following characteristics of the human mortality profile is an important task in the study of the evolution of aging. Among these characteristics are the mortality curve shape; the variability of mortality profile across generations; the presence of the postreproductive life period; and the modal age of death (i.e., the age when mortality is maximal).

### Technical progress as a factor that modifies the survival curves

**Figure 1:**
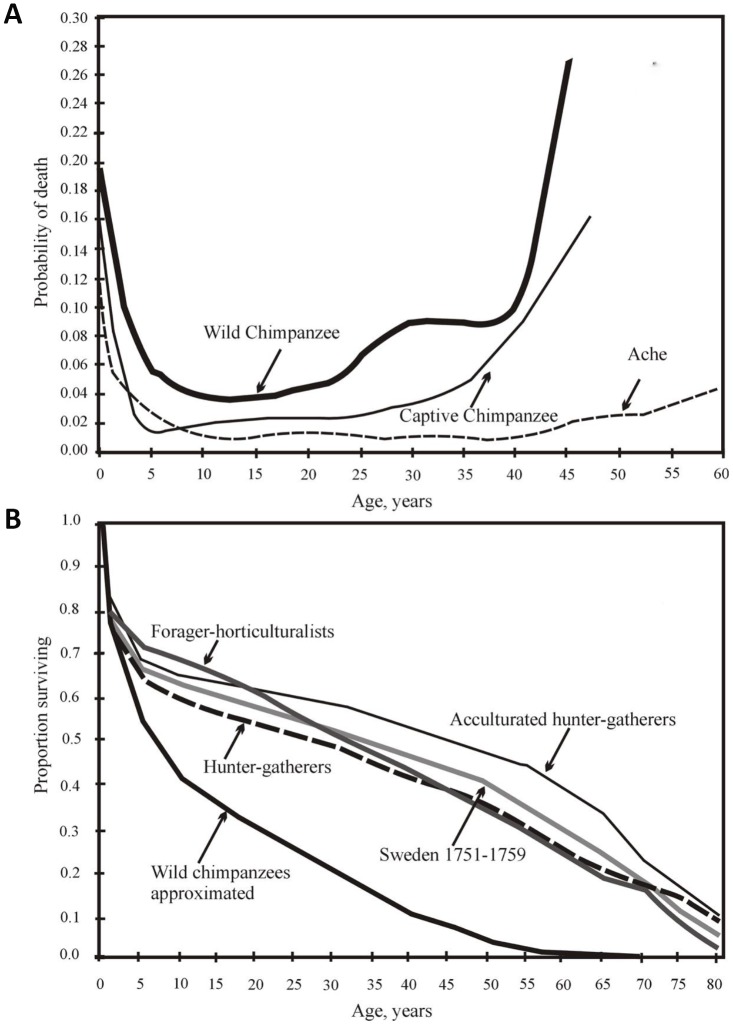
(**A**) from [44], with minor modifications. Yearly mortality of captive and wild chimpanzees [data from 43] and Ache Indians of Paraguay [32]). (**B**) from [47]. Survival of chimpanzees in the wild and the survival of various wild tribes of South America, Africa and Asia, and Swedes in 1751-1759.

The hunter-gatherers have a longer juvenile period of elevated mortality, and a longer adult LS as compared to chimpanzees (Figure 1A).

The mortality rates vary among different human populations and between different time periods, especially regarding the risk of sudden death. However, these differences are small from a comparative cross-species perspective. The mortality profile similarity among different preindustrial populations living in different conditions is evident (Figure 1B).

Dental and renal diseases and cerebrovascular pathologies, as well as uterine leiomyomas, are common age-related pathologies of humans and apes. It was previously believed that brain volume reduction, breast and prostate cancer, lung cancer and colorectal cancer, gout and Alzheimer’s disease are characteristic only of aging humans, not of apes [40]. However, the presence of the two classic damaging signs of Alzheimer’s disease (the accumulation of amyloid β and phosphorylation tau proteins with age) were demonstrated in old chimpanzees in 2017, indicating that this pathology is not specific to humans [41]. These signs had been previously described in *Oncorhynchus* during the spawning period [42].

### Life expectancy for humans, wild chimpanzees and captive chimpanzees

Captivity significantly increases the survival of chimpanzee infants and adolescents. The percentage of those who live to 15 years grows from 37% in the wild to 64% in captivity [43]. However, although the proportion of animals surviving to the age of 45 increases sevenfold (from 3% in the wild to 20% in captivity), it is still half as high as that for humans living in primitive conditions [44]. The difference between the chimpanzees and humans over 45 years old is even greater; the additional life expectancy (e_45_) for the captive chimpanzees is only 7 years, which is about one-third that of the e_45_ for humans. Thus, chimpanzees age much faster than humans and die earlier, even under external protection [44].

Forty-seven percent of the 15-year-old captive chimpanzees lived to the age of 47 years old, *the maximal observed age for wild chimpanzees* [44]. Thus, the aging (i.e., an increase in death probability with age) of a chimpanzee under wild conditions is largely determined by external causes and to a much lesser extent by internal causes. *H. sapiens* are generally characterized by a sharp decline in mortality from infancy to adolescence; mortality then remains very low up to the age of 40 years, after which it steadily increases (Figure 1A).

All the survival curves of Native American populations of *H. sapiens* proved to be at a considerable distance from the survival curve of wild chimpanzees. Additionally, despite all the differences (lifestyle, food, etc.), all human survival curves are extremely similar, intersecting at 80 years, that is, at the point corresponding to LS_95_ (Figure 1B).

This again demonstrates that LS_95_ is well suited for use in the analysis of the survival curves of *aging populations* (where the probability of the death of an individual increases with age): mice, rats, fruit flies, and nematodes [45]. However, LS_95_ is less convenient for the analysis of non-aging populations (by this criterion), for example, for *H. glaber* or higher plant populations [46].

The *life expectancies at birth (e_0_)* among different aboriginal tribes are quite similar even across the continents. The values of *e_0_*, in ascending order, are as follows: Hiwi (Venezuela) - 32 years, Hadza (Tanzania) – 33 years, !Kung (the Kalahari desert between Botswana and Namibia) and the XVIII century Swedish individuals – 35 years, Ache (Paraguay) – 38 years, Agta (Philippines) and Yanomamo (Venezuela) – 41 years, and Tsimane (Bolivia) – 42 years [47].

The probability of living to the age of 15 is also similar in all preagricultural tribes (from 55% in Hadza to 71% in Ache). The average value of this probability for all hunter-gatherers is 60%, while the average value for various groups of captive chimpanzees is 35% [36]. The 15-year-old hunter-gatherers will live, on average, 39 years more, whereas 15-year-old chimpanzees will live only 15 years more. Fifteen percent of all hunter-gatherers will live to 70 years and 5% to 80 years (i.e., up to the LS_95_ age) [36]. The maximal human LS is 122.5 years [48], almost twice the value for the chimpanzees (62 years in captivity [43]), which may partially be due to very different sample sizes for humans (many millions of individuals) and chimpanzees (thousands of individuals).

**Figure 2:**
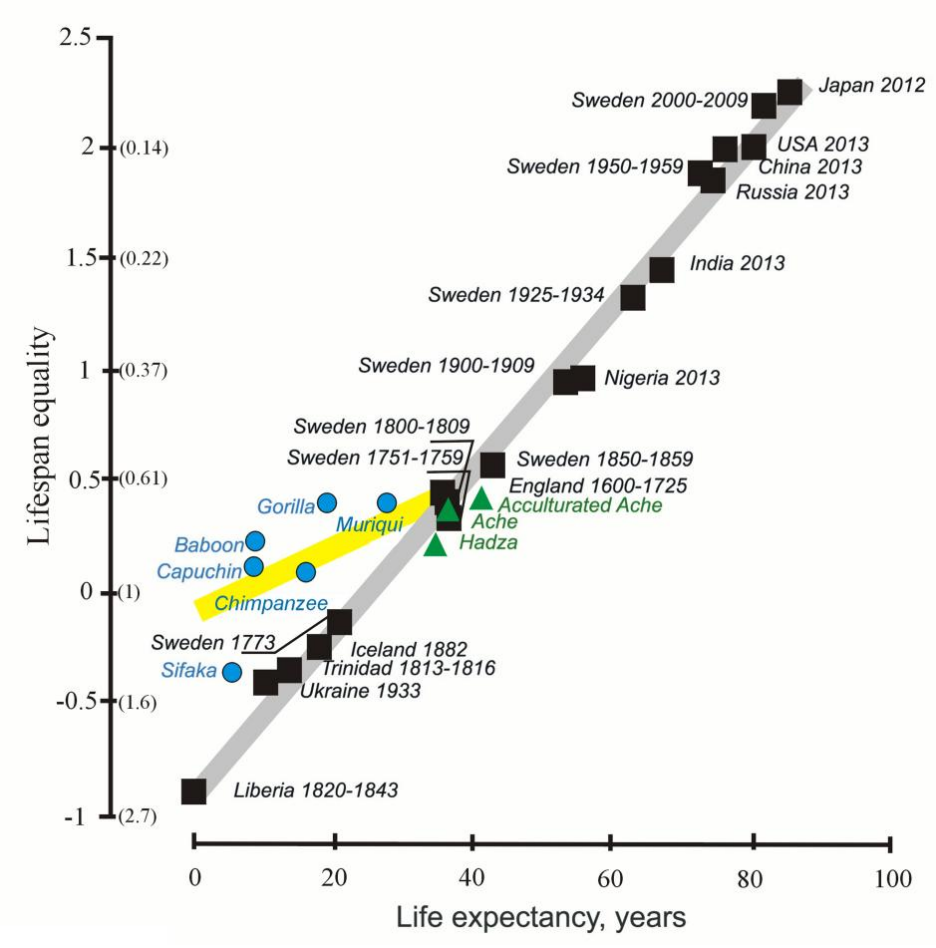
**The lifespan equality and life expectancy in humans (black and green) and non-human primates (blue) (from [49], with minor modifications).** The *y* axis shows lifespan equality, the log of the inverse of the Keyfitz’s entropy; corresponding values of the Keyftiz’s entropy are given in parentheses on the *y* axis.

The shape of the distribution of lifespans can be assessed in other ways, including the coefficient of variation of lifespan and Keyfitz’s entropy [49]. In 2012-2017 [34, 35], we suggested that the coefficient of variation can serve as an additional characteristic of the stability of mortality dynamics [35]. Similarly, [49], an equality indicator, based on the measuring of Keyfitz’s entropy, was proposed by Colchero et al. In 2016 [49]. Keyfitz’s entropy is given by the ratio e†/e_0_, where e† measures life expectancy lost due to the death: e† $=\int_{0}^{\infty }{\text{e}}_{\text{x}}\text{d}(\text{x})\text{dx}$ Keyfitz’s entropy is an indicator of lifespan inequality; the inverse of Keyfitz’s entropy is an indicator of lifespan equality (the log of the inverse is calculated [49]). A strong positive correlation of this value with life expectancy was shown both for different human populations living in different conditions (including Ache) and non-human primates [49] (according to [43, 44, 47]) (Figure 2). Low values of the indicator may result from high mortality in a particular historical period (for example, famine in Ukraine in the thirties of the 20^th^ century).

### Mortality rate doubling time (MRDT)

The rate at which the mortality rate doubles is the aging rate measure. It was reported that the mortality rate doubled every 7-8 years for a number of human populations with a very wide range of total mortality [47]. MRDT for the studied preagricultural populations is also mainly within the range of 6-10 years (for example, 6 years for Hadza, 7 years for Ache and 9 years for !Kung) [47].

Dependence on the initial mortality rate is an obvious disadvantage of this parameter. For instance, if the initial mortality rate is 0.01% per year, then it will soon double, while the mortality rate of up to 20% per year may not double throughout the lifetime of the cohort.

Therefore, it is hardly surprising that the MRDTs of various preagricultural tribes are so different (only 2.8 years for Hiwi and 12–18 years for Agta) [47].

**Figure 3:**
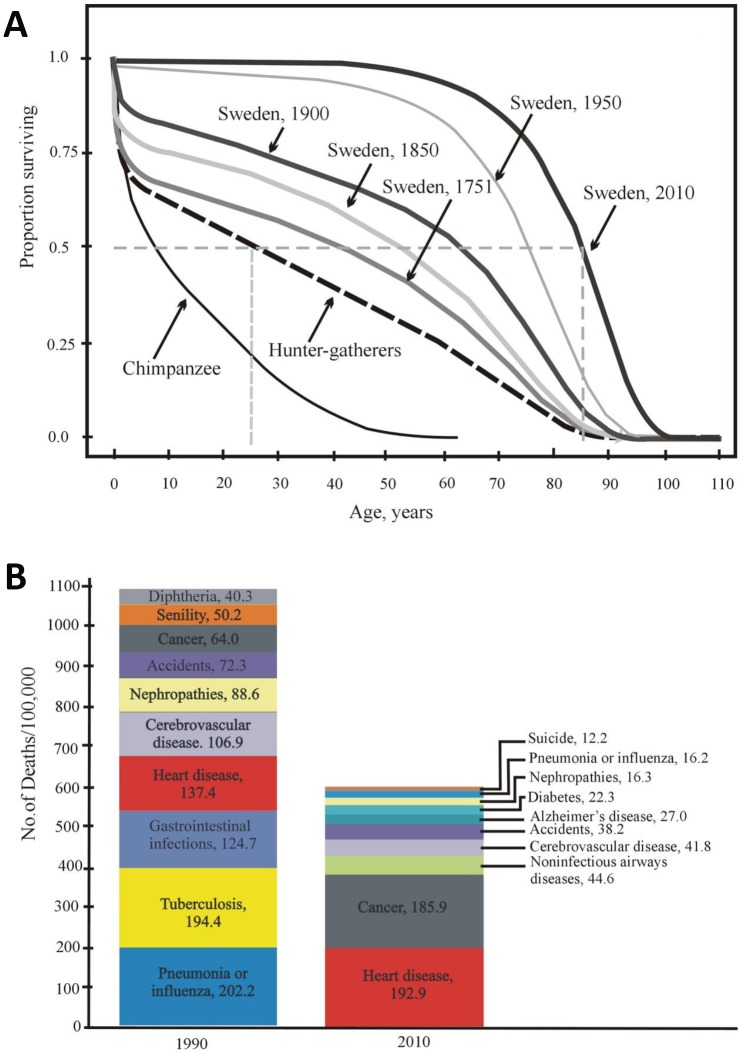
(**A**) Age-dependent survival of Swedish individuals (in 1751, 1850, 1900, 1950, and 2010) and of Ache Indians and chimpanzees (according to [50, 51] with modifications). (**B**) Mortality structure in the USA (10 main causes of death in 1900 and 2010) [52].

The age-dependent dynamics of the probability of death are surprisingly similar among different aboriginal tribes [47]. The mortality rate slows down to 1% per year at the age of 10 years, and then the mortality remains low to approximately 40 years old despite the obvious mortality rate acceleration with age [47]. The results obtained in the groups of hunter-gatherers (Figure 1B and 3A) are similar to the results from Sweden in 1751 (the post-Charles XII period). For example, the life expectancy at birth (e_0_) was 34 years, and e_45_ promised an additional 20 years. It turned out that at least one-fourth of the population would live for 15–20 years more after 45 years (without access to modern medical care, public sanitation, immunization, and a predictable food supply) [47].

Currently, Swedish and Japanese individuals practically show negligible mortality until 50-60 years old. Not more than 10% die over 55 years of life, and not more than 70% of the original population dies within 55-80 years of life. Over the last century, life expectancy at birth (e_0_) has increased in most countries and has exceeded 80 years in several countries [50]. Therefore, it is obvious that mortality in humans in developed countries has decreased so greatly at present that the difference between the hunter-gatherer mortality rate and the current mortality rate in developed countries today is much greater than the difference between hunter-gatherers and wild chimpanzees (Figure 3A).

Moreover, the reduction in human mortality is at the same level or exceeds that in other species, even in animals in captivity or subjected to life-extending mutations as well as laboratory selection for longer life. O. Burger notes that the observed plasticity of the age-related death risk contradicts the generally accepted theories of aging [50, 51], but it can be explained by the “theory of aging as part of a general program of ontogenesis” (see below).

The main part of the pronounced decline in mortality occurred in developed countries from 1900 to 2010 (approximately 4 human generations). Figure 3B shows the structure of mortality in the United States and its change over the aforementioned period from 1900 to 2010. Both earlier and now, diseases of the cardiovascular system contribute to the structure of mortality, taking first place in the number of deaths caused (32.2% in 2010 vs 12.5% in 1900) [52]. In addition, the role of neurodegenerative diseases deserves special attention. These pathologies are important, although they do not have an equally strong influence on the structure of mortality. For example, Alzheimer’s disease takes now sixth place on the list, i.e. 4.5% in 2010. However, neurodegenerative diseases have a very negative effect on the quality of life, socialization and self-sustainment ability of a human being. In fact, they greatly reduce the health span.

The change in survival curves of humans compared to chimpanzees occurs for two reasons: neoteny and very rapid technical progress. An analysis of time scales and survival curves allows us to separate these two causes. Thus, the evolution of neoteny requiring much more time may be responsible for the difference in the mortality curves of chimpanzees and hunter-gatherers, while technical progress is responsible for the great differences in the mortality curves of hunter-gatherers and Swedish individuals in the 20^th^ century.

## Aging subprograms as a part of ontogenetic program

The aging of an organism is manifested in an increase in the frequency of age-related diseases and, consequently, in an increasing probability of death [34]. The evolution of the aging phenomenon in Metazoa proceeded from potentially immortal forms. Potential immortality is an ancestral feature gradually lost in the course of evolution. At the same time, aging and death resulting from aging are facultative (rather than obligatory) features of Metazoa. The main principle of the evolution of aging is as follows: it is the substitution of external factors of death of non-aging Metazoa with internal ones programmed in the genome [17, 53]. If the probability of the death of an organism depended entirely on the degree of its age-related “wear”, then the mortality rate of multicellular organisms would increase with age regardless of the species position in the evolutionary tree. This has not been confirmed since the discovery of large differences in the mortality dynamics in different species (ascending, constant, descending, convex and concave mortality trajectories, both in the long-living and short-living species) [32, 33]. M.V. Skulachev and V.P. Skulachev [54, 55] pointed to this contradiction by proposing the acute phenoptosis concept. According to this concept, chronic phenoptosis (*slow* aging) can be finally substituted by a *fast* programmed death.

This concept originates from the assumption that septic shock is an example of pathology with signs of acute phenoptosis. Quick death (due to the massive release of special regulators to the blood) is apparently caused by the infected organism itself, presumably to remove the sick individual from the population in order to prevent an epidemic advance [17, 54, 60, 61]. Now it is suggested that aging (slow phenoptosis) results in such a crucial damage of physiological function that reproduction become impossible and the old organism is quickly killed by his own mechanism of acute phenoptosis to eliminate individual that cannot be involved in natural selection [54, 55]^2^.

### Anti-aging programs. Transcription factor Nrf2

A transcription factor called “Nuclear factor erythroid 2-related factor 2 (Nrf2)” seems to be a component of an anti-aging program [62], as well as the repair enzymes [63]. Nrf2 is thought to be a *guardian of the health span and a gatekeeper of species longevity* [62]. It induces the expression of genes encoding ~200 repairing and detoxifying enzymes, including the most powerful natural antioxidants [64]. Nrf2 signaling activity is positively correlated with LS [62]. The level of Nrf2 decreases in old mice [65–68].

Protein antagonists of Nrf2 (β-TrCP, KEAP1, Bach1 and c-Myc) [64, 69, 70] and mitochondrial ROS generation can be components of aging programs. The first three proteins are inhibited by ROS, which makes the situation even more complicated than the simple competition of anti-aging and aging programs.

### Keratin and heavy chain of myosin 16

The degradation of certain genes is one of the most important and “radical” evolutionary changes in the genome [71]. The human-specific loss of the heavy chain of myosin 16 (MYH16) gene occurred presumably due to a change in diet, which, in turn, reduced the dependence on powerful masticatory skeletal muscles. The loss (~2.4 million years ago) of MYH16 was probably a favorable adaptive change that released constraints on the increase in cranial volume and brain size [72]. In addition, the disappearance of the myosin heavy chain weakened the skeletal muscles of the limbs and reduced the overall weight of the body, although it also weakened the physical strength.

The transformation of a gene from a cluster of type I keratin genes (responsible for hair emergence during ontogenesis) into a nonfunctional pseudogene φhHaA (KRTHAP1) in humans was another relatively recent evolutionary event. Orthologs of this gene are functional in gorillas and chimpanzees[73].

### Neoteny in some invertebrates

Neoteny has been described in many invertebrates, such as insects (termites [74], mayflies, cicadas [75, 76], beetles [77–79]) and isopod crustaceans [80]. The *Turritopsis* jellyfish, one of the representatives of the cnidarians, has a unique opportunity *to* *revert*, in response to mechanical damage or other adverse changes in external conditions, to *an earlier state (polyp) from the stage of jellyfish that reproduce sexually* [81]. As a result, the rejuvenation of this organism occurs.

The issue of the presence of neoteny in ants (Hymenoptera, Formicidae) is disputable. A comparison of queens and gamergates^3^ may help in the search for ant neoteny [82]. Neoteny is formally absent in the worker ants in the majority of species since they never reach sexual maturity. At the same time, it is possible that the very appearance of the gamergates and intercastes in ants in the course of evolution proceeded along a neoteny path [83–85]. The intercastes include the individuals that morphologically differ from the typical winged members of their sex and resemble workers in some respects (the so-called ergatoid females and males). In particular, their wing muscles are not developed. The intercaste females are heterogeneous in their reproductive function: some are involved in egg laying, and others perform the functions of workers [86]. The ergatoid males breed but are not capable of flying. Such males mate in the nest [87].

The gamergates are found in different subfamilies of Formicidae and are especially common in the most ancient Ponerinae subfamily [88–91]. The gamergates have no distinctive external features compared to workers and reproductive females. The gamergates develop from the same young individuals as the workers, and their ability to lay eggs is determined by interactions with other individuals [92, 93].

It is also possible that the caste of workers has been formed not through the development of neoteny but as a separate ontogenetic trajectory since many morphophysiological differences between the individuals in the families of ants are caused by phenotypic plasticity rather than by hereditary variation. In this regard, it is interesting that the size of the brain in the egg-laying gamergates can be reduced compared to the working females. In *Harpegnathos* *saltator,* the brain volume of gamergates is only 74% that of workers [94]. It is noteworthy that the *Harpegnathos* workers who stand out among the ants with very large eyes have extremely developed optical brain lobes, whereas just this part of the brain undergoes a strong reduction in gamergates that spend their whole life in the nest. The authors suggest that the nutrient-rich brain material is expended for egg production. A similar process caused by the lysis of the wing muscles in the fertilized ant females was described by Janet [95].

### Neoteny in vertebrates

Among vertebrates, the Mexican salamander (*Ambystoma mexicanum*) is a classic example of neoteny; this animal can reproduce at the larval stage (axolotl). Its LS is more than 32 years. The transformation of an axolotl into an adult *Ambystoma* can be achieved by the addition of the hormone thyroxin. Moreover, *A. tigrinum* can transformed to adult *Ambystoma* even spontaneously under conditions which can be suspected to cause general disturbance in the central nervous system, e.g., when animals are caught and taken into the laboratory, or during periods of high temperature [96]. Among the caudate amphibians, there are species, for example, the European proteus (*Proteus anguinus*), whose adult form has not been described (obligate neoteny). Their maximal reported lifespan (in captivity) is 69 years [97], while the LS estimated value reaches 103 years [98]. A salamander resembling the axolotl but having no adult state (*Necturus maculosus*) lives for more than 29 years and survives only in water [99]. Neoteny has also been found in ray-finned fish [100, 101], lobe-finned lungfish (closely related to the Tetrapoda) [102] and some Passeriformes [103].

Neoteny in mammals, in addition to humans and *H. glaber*, was hypothesized for baleen whales [104] and toothed whales (dolphins) [105] as well as for dogs (“behavioral neoteny”) [106].

### Naked mole rat neoteny

The *H. glaber*, a rodent species with an LS unusually high for its weight, has pronounced neotenic traits (for reviews, see [13, 26]). Senescence-related traits, such as muscular and fatty weight reduction, lipofuscin deposition in tissues, and cataracts, start to develop only by approximately 30 years of age in *H. glaber* [107, 108].

The neoteny of the lungs in *H. glaber* does not manifest itself as decisively as the neoteny of the hair: the hair completely disappears, and the lungs remain underdeveloped.

The DNA fingerprinting method demonstrated that *H. glaber* individuals within colonies were genetically almost monomorphic (coefficients of band sharing estimated from DNA fingerprints range from 0.93 to 0.99) [109]. *H. glaber* has a low level of heterozygosity (1.87 million heterozygous single-nucleotide polymorphisms per diploid genome or ~0.7 heterozygous sites per thousand nucleotides). Similarly, humans have significantly lower heterozygosity than mice and rats [110]. The morphological differences between individuals are probably formed mostly by epigenetic mechanisms. Other peculiar features of the *H. glaber* genome have also been demonstrated. For example, at least one of the two mammalian characteristic genes, *Mtnr1a* and *Mtnr1b*, is nonfunctional [110]. *Mtnr1a* and *Mtnr1b* are receptors of melatonin – a hormone responsible for biorhythm [110, 111]. Despite the presence of a corresponding gene, the expression of the last enzyme of the melatonin synthesis pathway (N-acetyl serotonin-O-methyltransferase) is suppressed in naked mole rats, at least in the brain, liver and kidneys [110, 112].

Moreover, the epiphysis has not been found in *H. glaber* ([113], single-animal study, confirmed by V. Manskikh in our group). *H. glaber* does not respond significantly to the increased *Fos* gene expression level in the suprachiasmatic nucleus of the hypothalamus in response to increased illumination, which is different from the response of other visually impaired fossorial species, such as the common mole rat *Cryptomys hottentotus* [114, 115].

### L. Bolk’s hypothesis: *pro and contra*

As noted above, two factors may account for the LS increase: 1) improved living conditions and 2) the retardation of switching on an aging program, e.g., the disappearance with age of mitochondrial mild depolarization [116]. Humans are traditionally compared to chimpanzees, although these two species have undergone separate evolution under different conditions for a long time. As a result, the modern structure of the human body is a mosaic with many characteristics that develop more slowly in humans than in chimpanzees, while other features, on the contrary, develop faster [117]. For example, the high growth rate of the brain during ontogenesis in human embryos persists until birth, while in chimpanzees, the growth rate of the brain begins to decline at the prenatal stage of ontogenesis [118, 119]. The brain of *H. sapiens* grows for approximately 15 years after birth, reaches 95% of the adult size between 7 and 11 years, and only then completes the final 5% of growth [120]. The chimpanzee brain volume reaches that of an adult by the age of 5 years [121]. Similarly, the postnatal brain maturation of *H. glaber* takes four times longer than that of a mouse, despite a more mature brain at birth [122].

In 1871, Darwin pointed to the similarity of the embryos of monkeys and humans, while their adults are much less similar [123]. In the 1920s, L. Bolk was the first to develop in detail the idea of the presence of numerous neotenic (pedomorphic) features in adult humans. He compiled a detailed list of the neotenic traits of humans, which was subsequently supplemented by other authors. In addition to a long period of infantile dependence and growth, greater LS and larger brain weight, there are other numerous neotenic differences between adult humans and other primates. Among them are the features of the skull [brachycephaly, orthognathia (the absence of a protruding muzzle)]; the absence of skull crests; skull bone thinness; a small amount of change in the skull structure from birth to old age; the absence of superciliary arches; certain variations in the structure of the teeth and cranial sutures; the position of the eye sockets below the cranial cavity; the central position of the occipital foramen (migrates backwards during ontogenesis in most primates); small teeth; later teething; some other infantile features of the skeleton (e.g., pelvis shape, longer legs compared to the arms, short limbs compared to the body size, inability for full thumb rotation, the absence of the baculum); the shape of the outer ear, epicanthic fold (skin fold at the upper eyelid), and labia; and a ventrally directed position of the genital canal in women.

Some traits almost disappeared in humans (e.g., body hair and skin pigmentation loss in some populations) [20, 21, 124–128]. The same is true for some skull structures [129]. Humans have sacrificed part of their physical strength in exchange for complex of brain activity (for example, a gorilla is 15 times stronger than a human being; [117]). Such unique human features as bipedality and the reduction in the size of canine teeth appeared before the big brain and stone tool making, presumably due to the transition to monogamy and the increasing contribution of fathers to the care of their offspring [130–132].

S. Gould [124] and, later, J. Verhulst [127] revised Bolk’s theory without refuting it. Verhulst indicated that, in addition to neotenic traits, a number of human morphological features, which are usually considered specializations caused by natural selection, are examples of hypermorphosis (i.e., changes in body proportions caused by the simple prolongation of ontogenesis [124, 127]). As the examples of this, the author mentioned the flattened chest of a human being and some other proportions of his or her physique. As an example of a particularly important hypermorphosis, Verhulst mentioned the structure of the larynx being different in adult humans from the larynx structure of other primates and infants [127]. This hypermorphosis is assumed to have been very important for the formation and socialization of a human being [132].

Strictly speaking, hypermorphosis can be attributed to a special case of neoteny. The situation is rather complicated due to the differences in neoteny levels of different traits and mosaic ontogenesis. In this case, one can rely on the number of characters, the development of which requires significantly more time in the neotenic species than in related non-neotenic species. If this number is much greater than the number of traits that develop faster than usual, then this is neoteny. However, a more attractive criterion of neoteny might be a *functional* analysis of each trait. For example, a strong argument in favor of neoteny can be a situation where a trait is directly involved to higher life span. For example, in our group, it was recently discovered that mild mitochondrial depolarization, which prevents the formation of mitochondrial ROS, in mice disappears by the age of 2.5 years, and in naked mole rats, depolarization persists for decades [116]. Strong retardation of depolarization and ROS production in naked mole rat prevents age-related protein carbonylation in these animals and, hence, increases their LS.

The study of the transcriptome in the prefrontal cortex of humans, chimpanzees and rhesus monkeys has revealed that it undergoes heavy reconstruction in the postnatal period, which proceeds slower in humans than in apes. In particular, the maximal expression of the genes responsible for synapse formation in the prefrontal cortex in chimpanzees and macaques has been observed at the age of one year and at the age of five years in humans [36]. The period of pregnancy in humans takes more time [22, 133], and the weight gain rate during the first five years after birth is also higher (2.6 kg/year vs 1.6 kg/year for chimpanzees), although this parameter turns almost the opposite over the next 5 years [36].

In general, the difficulty in the interpretation of the role of LS-affecting genes is because the ratio of the contribution of genes and nongenetic components to the variability of the characteristics of each species is not a constant value but rather depends on circumstances. It is obvious that the same allele can reduce the LS of animals in adverse conditions but may not affect the LS in good conditions. The LS-affecting genes in the hunter-gatherers may have no effect on the LS of the urban Japanese population. For example, a gene that affects the joints or any other important organs involved in heavy physical work will not affect the observed aging rate in a society carrying out a small amount of such work.

## CONCLUSION

There is still no agreement among gerontologists as to the main aging-related issue: whether it is an accidental accumulation of damage in the organism or a result of the operation of a specially evolved program. In other words, it is still not clear whether aging is an inevitable phenomenon that is uncontrollable by organisms or whether it is a facultative adaptation that enhances the adaptive ability of species, i.e., their evolvability.

The undoubted neoteny of *H. glaber* has been described in the last three years: up to now, 58 neotenic traits were found in this species [13, 24–26, 134]. The slowing down of such a large number of developmental processes clearly shows that the longevity of *H. glaber* is a result of a general slowdown of late ontogenesis.

In 2017-18, the increase in acute phenoptosis was directly demonstrated in the nematode *C. elegans.* Phenoptosis starts as early as the first day of adult life due to the initiation of two special programs. The first program prevents the organism from being protected against oxidative, thermal, toxic and other stress types, and the second is a program of the autolysis of the intestine. Compounds and energy saved by these two programs are used for the biosynthesis of the yolk of eggs laid by the worm. The first program is activated by blocking specific histone demethylation [12, 27, 28], and the second is activated through insulin stimulation of autophagosomes that attack intestinal cells [29].

The evolutionary changes in humans compared to other primates have the following distinguishing characteristics: large brain, exceptionally large LS, high paternal investment in offspring, and the role of older individuals as helpers in upbringing the children [47]. The large brain is associated with a change in psychological characteristics: enhanced learning and cognition. Even human sleep is shorter, deeper, and has more rapid eye movement phases than that in other primates. Supposedly, the selection pressure in the direction of the reduction in sleep duration and its “quality” improvement were activated in the early stages of human evolution due to the change in the ecological niche and the development of overnight stays on the ground and not in the tree branches [135].

The evolution of these life history characteristics and extremely high intelligence was probably related to some degree to the dietary transition to high-quality, solid and hard-to-get food resources. Improvements in living conditions were due to technical progress (improved quality of food, medical services, industrial goods, etc.) and other evolutionary adaptations: cephalization index, sociality, and postreproductive LS have important effects on the survival curves in humans. A similar effect with respect to the reproductive individuals of *H. glaber* is caused by the fact that a single breeding female and one or two breeding males can be protected by hundreds of subordinates. Apparently, such protection also enhanced the selection against harmful mutations with late effects, which leads to a slowdown in aging, that is, to the activation of anti-aging programs [63, 116, 136].

In humans, technical progress leads to a sharp decrease in infant mortality and an increase in life expectancy, especially in comparison to wild chimpanzees [51]. Despite the huge variation in the LS of various human populations, starting with preagricultural tribes and ending with the urban population in the developed countries, the differences between their survival curves are still smaller than those between the preagricultural human populations and the chimpanzees living in the wild. This relationship can be explained by the fact that neoteny prolongs LS and health span.

The improvement in living conditions by new medicinal products, such as mitochondria-targeted antioxidants, could be effective in prolonging the average lifespan so that they can be considered not just as age-related disease treatment drugs but also as “true” anti-aging drugs. Such products may suppress chronic and acute phenoptosis processes. The example of such medicine is a mitochondria-addressed antioxidant SkQ developed at the Belozersky Institute, Moscow State University [55, 137–145]. Rapamycin, which seems to cause aging retardation and an increase in LS, appears to act in the same direction [146–149] due to the suppression of chronic phenoptosis. Metformin also causes a significant increase in LS and the retardation of aging, not only due to its properties as an antidiabetic drug [150–152] but also as an inhibitor of the aging program. It is essential that both SkQ and metformin specifically inhibit ROS generation in mitochondria at the beginning of the respiratory chain, where SkQ is effective at a thousand times lower concentration than metformin.

The retardation of the aging process in the human body with special drugs is a promising approach to extend the health span. Such an approach appears probable because aging retardation in some mammals is already achieved through neoteny, a natural physiological phenomenon.

## Footnotes

2 It is often assumed that the traits appearing in the postreproductive period are not subject to natural selection [1, 4, 56]. However, in certain cases the skills useful for the population and acquired in the postreproductive period by some animals (especially social ones) are transferred, via social learning, to the individuals still capable of reproduction, thus increasing their fitness and, in general, increasing the resilience of the population and hence adaptations to changes of the external environment [57–59]. If longevity is favored by selection, the special protective and repair systems might evolve that slow down chronic phenoptosis. Since LS is a stable species-specific characteristic, similar to body size or fecundity, the life duration should somehow be programmed in the genome [3, 8, 42].

3 Gamergates – worker ants able to reproduce sexually.
